# Stranger Months: How SARS-CoV-2, Fear of Contagion, and Lockdown Measures Impacted Attendance and Clinical Activity During February and March 2020 at an Urban Emergency Department in Milan

**DOI:** 10.1017/dmp.2020.265

**Published:** 2020-07-27

**Authors:** Stefano Franchini, Marzia Spessot, Giovanni Landoni, Cecilia Piani, Chiara Cappelletti, Federica Mariani, Simona Mauri, Maria Vittoria Taglietti, Manuela Fortunato, Federico Furlan, Barbara Guglielmi, Eleonora Setti, Davide Di Napoli, Giovanni Borghi, Federico Pascucci, George Ujlaki-Formenti, Riccardo Sannicandro, Matteo Moro, Sergio Colombo, Lorenzo Dagna, Antonella Castagna, Moreno Tresoldi, Patrizia Rovere-Querini, Alberto Ambrosio, Fabio Ciceri, Alberto Zangrillo, Michele Carlucci, Roberto Faccincani

**Affiliations:** IRCCS San Raffaele Scientific Institute, Milano; Vita-Salute San Raffaele University, Milano

**Keywords:** COVID-19, emergency department, direct and indirect impact

## Abstract

**Objectives:**

An unprecedented wave of patients with acute respiratory failure due to severe acute respiratory syndrome coronavirus 2 (SARS-CoV-2) disease 2019 (COVID-19) hit emergency departments (EDs) in Lombardy, starting in the second half of February 2020. This study describes the direct and indirect impacts of the SARS-CoV-2 outbreak on an urban major-hospital ED.

**Methods:**

Data regarding all patients diagnosed with COVID-19 presenting from February 1 to March 31, 2020, were prospectively collected, while data regarding non-COVID patients presenting within the same period in 2019 were retrospectively retrieved.

**Results:**

ED attendance dropped by 37% in 2020. Two-thirds of this reduction occurred early after the identification of the first autochthonous COVID-19 case in Lombardy, before lockdown measures were enforced. Hospital admissions of non-COVID patients fell by 26%. During the peak of COVID-19 attendance, the ED faced an extraordinary increase in: patients needing oxygen (+239%) or noninvasive ventilation (+725%), transfers to the intensive care unit (+57%), and in-hospital mortality (+309%), compared with the same period in 2019.

**Conclusions:**

The COVID-19 outbreak determined an unprecedented upsurge in respiratory failure cases and mortality. Fear of contagion triggered a spontaneous, marked reduction of ED attendance, and, presumably, some as yet unknown quantity of missed or delayed diagnoses for conditions other than COVID-19.

On February 20, 2020, the first autochthonous case of COVID-19 in Italy was identified in Lombardy, a region of northern Italy. In the following weeks, the country witnessed a steep increase in COVID-19 cases and COVID-19-related deaths.^1^ As of March 31, 2020 (the end of the period of this study), Italy was the second most affected country in the world (after the United States) with 105,792 total confirmed cases and 12,428 COVID-19-related deaths.^2^ Of those cases, 41% (and 58% of the deaths) were observed in Lombardy (which accounts for approximately 17% of the Italian population), by far the most affected Italian region.

The direct health impact of a pandemic can be catastrophic, and the indirect impact, driven by fear or depletion of resources, can further increase morbidity and mortality.^3^ At present, data describing the consequences of the COVID-19 outbreak on ED attendance and resource use are lacking.

This study aims to evaluate the direct and indirect impacts of the COVID-19 outbreak on an urban emergency department (ED). The primary aim is to assess attendance flows, clinical activity, and mortality generated by patients attending the ED of San Raffaele Hospital (OSR), a tertiary care 1350-bed academic hospital in Milan, capital of the Lombardy region, during the first 2 mo of the Italian COVID-19 outbreak, ie, from February 1 to March 31, 2020, and to compare this 60-d period with the corresponding period of 2019. We also assessed the time trends of such data and their relation with some crucial local epidemiological events that marked the evolution of the epidemic in Lombardy.

## METHODS

This is an observational prospective-retrospective study with descriptive purposes. This series is part of the COVID-19 institutional clinical-biological cohort assessing patients with COVID-19 (Covid-BioB, ClinicalTrials.gov NCT04318366) at OSR. Data regarding patients diagnosed with COVID-19 evaluated from February 25 on were prospectively collected, as part of the Covid-BioB cohort. The study was approved by the Hospital Ethics Committee (protocol number 34/int/2020). Data pertaining to the non-COVID19 2020 and 2019 patients were retrospectively retrieved by electronic chart review.

During the February 1 to March 31 2020 timespan, 2 relevant events occurred: (i) on February 20, the first autochthonous COVID-19 patient was identified in Lombardy; (ii) on March 8, the Italian Government issued a decree enforcing strict lockdown measures involving the whole Lombardy region.^4^ According to these 2 events, we segmented the study period in 3 phases: the first phase (P1-2020) ranging from February 1 to February 20, the second phase (P2-2020) from February 21 to March 8, and the third phase (P3-2020) from March 9 to March 31. For comparison, we identified 3 corresponding phases in 2019: from February 1 to February 20 (P1-2019), from February 21 to March 9 (P2-2019), and from March 10 to April 1 (P3-2019), to account for 2020 being a leap year.

All patients presenting to the ED from February 1 to April 1, 2019, and from February 1 to March 31, 2020, were included in the analysis. The OSR ED treats approximatively 70,000 patients a year (approximately 200 a d). Starting from February 26, 2020, any patient presenting to the OSR ED with fever, dyspnea, cough, or other flu-like symptoms was placed in the newly created “Respiratory Medicine” area, reserved for suspected COVID-19 cases.

We collected the following data: age, sex, modality of arrival to the ED, chief complaint at triage, specialty area assigned to the patient by the triage nurse (respiratory medicine for suspected COVID-19 cases, general medicine, surgery, gynecology/obstetrics, orthopedics, ophthalmology, or pediatrics), need for oxygen therapy, need for noninvasive ventilation (NIV), confirmed final diagnosis of COVID-19, admission to the wards or the intensive care unit (ICU), ED mortality, and in-hospital mortality. Diagnosis of COVID-19 was defined as a SARS-CoV-2 positive real-time reverse-transcriptase polymerase chain reaction from a nasal and/or throat swab together with signs, symptoms, or radiological findings suggestive of COVID-19.

We retrieved, from official national sources,^5^ epidemiologic data pertaining to the trends of the SARS-CoV-2 epidemic in Lombardy within the 2020 period included in this study (confirmed cases and COVID-19-related deaths).

Data were managed by Microsoft Excel Ver. 16.0. Statistical analyses were performed with IBM SPSS Statistics Ver. 20 and with Prism 8 for Windows. The number of the major events considered in this study (namely, number of patients attending the ED, number of patients needing oxygen therapy, number of patients requiring NIV, patients admitted to hospital from ED, patients admitted to ICU from ED, patients deceased during hospital stay, number of patients with a confirmed diagnosis of COVID-19) were grouped for the 2020 and 2019 time windows, and further segmented into the 3 above-mentioned phases. Data were analyzed as daily absolute frequencies of each event. Because the general ED attendance dropped significantly in 2020, those events were also analyzed as mean proportions of the total attendances (categorical values). The Mann-Whitney U-test was used to compare the differences in the distributions of daily frequencies or of other continuous variables (eg, age). The proportions of the total attendances (expressed as percentages) referring to specific periods were compared using the chi-square test.

## RESULTS

A total of 7824 patients were admitted in 2020, compared with 12,422 in 2019 (–37%; *P* < 0.0001), including 641 confirmed COVID-19 patients for a total of 658 COVID-19 visits (8.4%). Table 1 reports the general features of the included cases, grouped by year of presentation and diagnosis of COVID-19. Compared with 2020 non-COVID patients, COVID-19 patients (Table 1) were predominantly male (*P* < 0.0001), older (*P* < 0.0001), and showed markedly higher admission (*P* < 0.0001) and mortality rates (*P* < 0.0001).

**TABLE 1:** General Characteristics of Patients Attending the ED in the 2020 and 2019 60-Day Periods, and Relative Changes Occurred in 2020 With Respect to 2019

	2020					***P Value (2020 vs 2019 overall)***
	Non-COVID	COVID	***P Value (COVID vs Non COVID)***	Overall	2019	
Mean age	43.4	61.4	***<0***.***0001§***	45.7	42.9	***<0***.***0001§***
Mean age of admitted patients	56.3	64.1	***<0***.***0001§***	58.1	54.4	***<0.0001§***
Female:male ratio	1.1	0.5	***<0.0001‡***	1.0	1.1	***0***.***014‡***
Female:male ratio of admitted patients	1.2	0.4	***<0.0001‡***	0.9	1.1	*0.061‡*
Admission rate	19.1%^	78.1%	***<0.0001‡***	23.5%	14.9%^	***<0***.***0001‡***
In-hospital mortality	147	114	***0.014§***	261	110	***<0***.***0001§***
Deceased during ED stay	26	7	***0.002§***	33	24	*0.574*
Deceased after admission	121	107	*0.137§*	224	86	***<0***.***0001§***
Total mortality rate	2.1%*	17.3%	***<0***.***0001‡***	3.4%	0.9%*	***<0***.***0001‡***
Total attendances	**7166**	**658**	***<0***.***0001§***	**7824**	**12422**	***<0***.***0001§***

§ *P* value obtained by Mann-Whitney U test.

‡ *P* value obtained by chi square test.

^ *P* < 0.0001 comparing 2019 patients with 2020 non-COVID patients, as obtained by chi square test.

* *P* < 0.0001 comparing 2019 patients with 2020 non-COVID patients, as obtained by chi square test.

**FIGURE 1:**
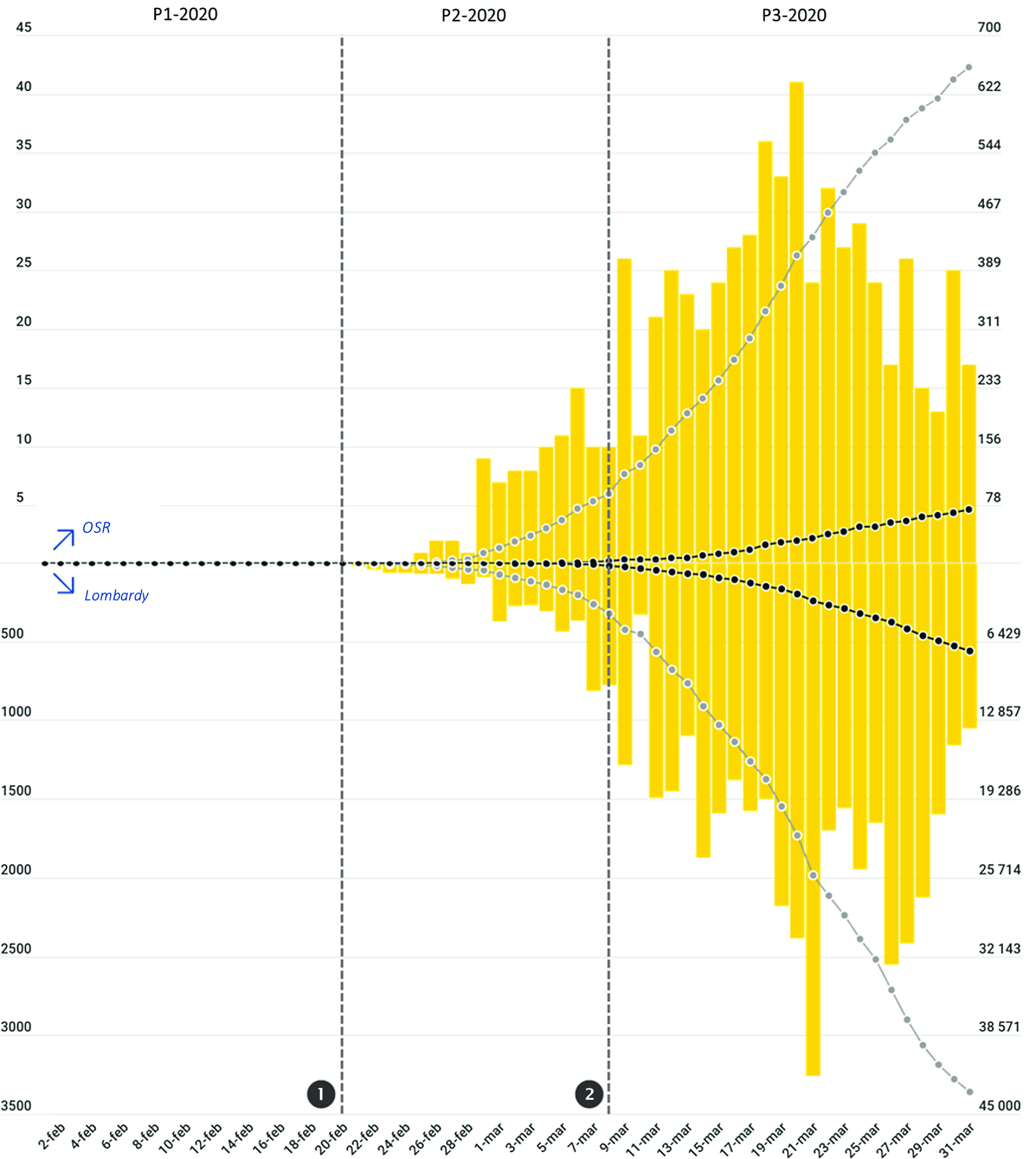
Trends of Patient Attendance at the ED of OSR in 2019 (Gray Line) and in 2020 (Black Line). Vertical dotted gray lines represent the 2 major events that occurred during the period included in the analysis (❶ the first patient infected in Italy is identified in Lombardy; ❷ lockdown measures are enforced to the whole population of Lombardy), marking the segmentation of the 2020 period into the 3 phases P1-2020, P2-2020, P3-2020. Horizontal dotted lines (identified by lowercase letters) represent the mean daily attendance value for each phase in 2020 (in black) and 2019 (in gray); a = P1-2019 mean daily attendance (212 patients/d); b = P2-2019 mean daily attendance (203 patients/d); c = P3-2019 mean daily attendance (205 patients/d); d = P1-2020 mean daily attendance (205 patients/d); e = P2-2020 mean daily attendance (118 patients/d); f = P3-2020 mean daily attendance (74 patients/d). Mean daily attendance dropped from 205/d in P1-2020 to 74/d in P3-2020. Two-thirds (66%) of that reduction occurred at the beginning of P1-2020, immediately after ❶, while the remaining 34% occurred after ❷.

Figure 1 depicts the trends of the confirmed COVID-19 cases and of COVID-19-related deaths recorded in Lombardy and at OSR. At the OSR ED, a short initial period of relatively gradual increase in the number of daily new cases of COVID-19 was followed by a steep rise beginning on March 9 that reached a peak on March 20. Starting on March 21, 13 d after the beginning of the lockdown, the number of daily new cases began to steadily diminish.

**FIGURE 2:**
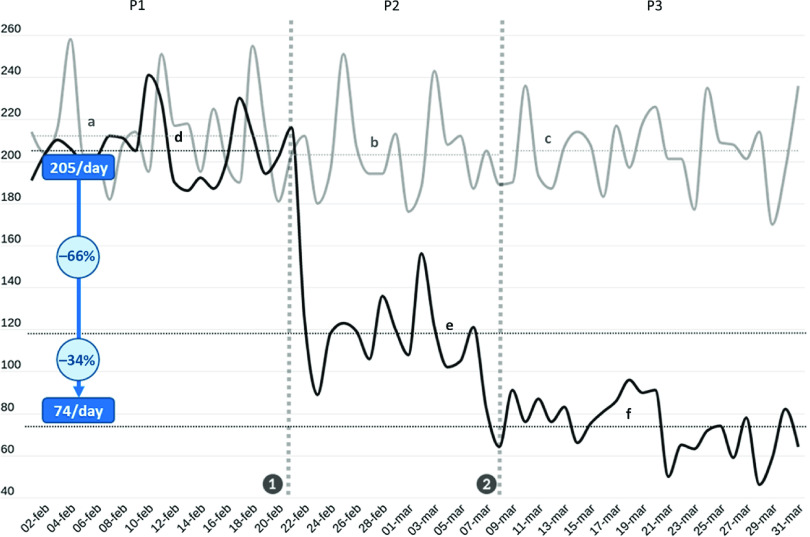
Trends of Patient Attendance at the ED of OSR in 2019 (Gray Line) and in 2020 (Black Line). Vertical dotted gray lines represent the 2 major events that occurred during the period included in the analysis (❶ the first patient infected in Italy is identified in Lombardy; ❷ lockdown measures are enforced to the whole population of Lombardy), marking the segmentation of the 2020 period into the 3 phases P1-2020, P2-2020, P3-2020. Horizontal dotted lines (identified by lowercase letters) represent the mean daily attendance value for each phase in 2020 (in black) and 2019 (in gray); a = P1-2019 mean daily attendance (212 patients/d); b = P2-2019 mean daily attendance (203 patients/d); c = P3-2019 mean daily attendance (205 patients/d); d = P1-2020 mean daily attendance (118 patients/d); e = P2-2020 mean daily attendance; f = P3-2020 mean daily attendance (74 patients/d). Mean daily attendance dropped from 205/d in P1-2020 to 74/d in P3-2020. Two third (66%) of that reduction occurred at the beginning of P1-2020, immediately after ❶, while the remaining 34% occurred after ❷.

The 37% reduction in overall ED attendance observed in 2020 (Table 2; Figure 2), was concentrated in phases P2-2020 (–41.8%; *P* < 0.0001) and P3-2020 (–63.8%; *P* < 0.0001). In fact, during P1-2020, before the identification of the first COVID-19 patient infected in Italy, the patient flow was approximately equal to that observed in P1-2019 (ie, –3.3%; *P* = 0.234). Of interest, two-thirds of the total reduction observed between P1-2020 and P3-2020 occurred immediately at the onset of P2-2020, before the lockdown measures were put in place, while the remaining 34% of reduction came after the lockdown. The falling trend in visits involved all areas of the ED. The most striking reductions occurred during the P3-2020 phase (compared with P3-2019) in the pediatric area (−95.3%; *P* < 0.0001), in the orthopedic area (−92.4%; *P* < 0.0001), in the ophthalmologic area (-89.7%; *P* < 0.0001), and in the surgical area (-80.6%; *P* < 0.0001).

**TABLE 2:** Absolute and Percentage Changes of ED Attendance in 2020 Compared With 2019, Stratified by Phase and Grouped by Specialty Area^a^

		P1			P2			P3			Total		
		Attendances	% Change	***P Value***	Attendances	% Change	***P Value***	Attendances	% Change	***P Value***	Attendances	% Change	***P Value***
**MED**	**2020**	1396	10.0%	***0.0108***	875	-15.5%	***0.0065***	977	-28.5%	***<0.0001***	**3248**	**-11.5%**	***0.0051***
	**2019**	1269			1035			1366			**3670**		
**SURG**	**2020**	913	-3.2%	*0.2223*	461	-44.6%	***<0.0001***	225	-80.6%	***<0.0001***	**1599**	**-45.5%**	***<0.0001***
	**2019**	943			832			1161			**2936**		
**OPHT**	**2020**	135	-12.9%	*0.5912*	50	-61.8%	***0.027***	21	-89.7%	***0.001***	**206**	**-57.9%**	***0.0002***
	**2019**	155			131			203			**489**		
**ORTH**	**2020**	551	-10.8%	*0.1181*	231	-57.5%	***<0.0001***	65	-92.4%	***<0.0001***	**847**	**-57.9%**	***<0.0001***
	**2019**	618			543			850			**2011**		
**OB/GYN**	**2020**	351	-22.0%	***0.0055***	228	-28.8%	***0.0024***	225	-48.9%	***<0.0001***	**804**	**-33.6%**	***<0.0001***
	**2019**	450			320			440			**1210**		
**PED**	**2020**	743	1.1%	*0.984*	161	-70.3%	***<0.0001***	29	-95.3%	***<0.0001***	**933**	**-50.9%**	***<0.0001***
	**2019**	735			543			623			**1901**		
**OVERALL**	**2020**	4103	-3.3%	*0.234*	2011	-41.8%	***<0.0001***	1710	-63.8%	***<0.0001***	**7824**	**-37.0%**	***<0.0001***
	**2019**	4243			3458			4721			**12422**		

a Differences in absolute daily attendance distribution are compared using the Mann-Whitney U test.

Abbreviations: MED, general medicine area (data regarding patients assigned to the “Respiratory Medicine” area -see text- are included in MED); SURG, surgical area; OPHT, ophthalmologic area; ORTH, orthopedic area; OB/GYN, obstetrical and gynecological area; PED, pediatric area; P, phase.

A comparison of patients’ chief complaints at ED triage between 2019 and 2020 demonstrates profound changes during the COVID-19 outbreak (Supplemental Table S1 and Table S2). In line with the massive general reduction of ED attendance, all complaints showed diminished frequencies, with the exception of dyspnea (+69%), fever (+51.5%), and cough (+24.1%). The greatest reductions were observed for common complaints often associated with low-severity clinical pictures, such as skin rashes (−59.6%), ocular symptoms (−58.3%), superior or inferior limb pain (respectively, −57.7% and −50.9%), lumbar pain (−53.6%), trauma (including minor trauma, −54.4%), diarrhea (−54.1%), and headache (−51.8%).

**FIGURE 3:**
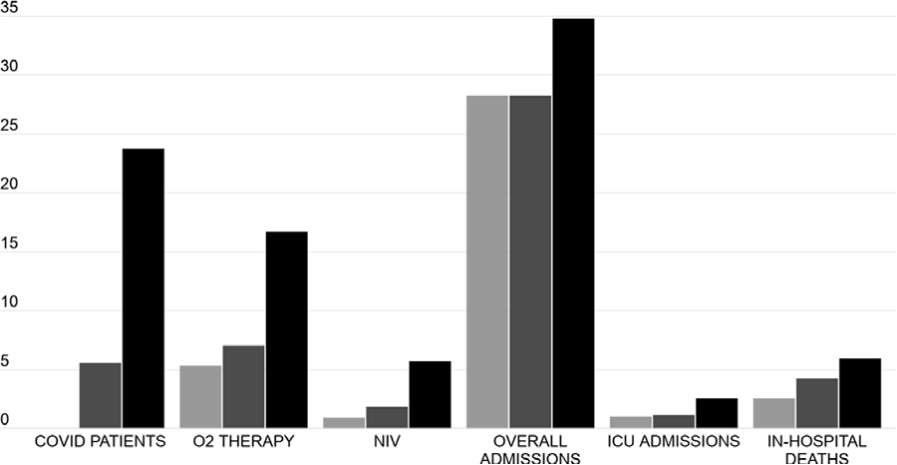
Description of Major Events. The columns represent the mean daily frequency of the observed major clinical events, namely, new COVID-19 cases, patient needing oxygen therapy or NIV, total admissions, ICU admissions, in-hospital deaths. Light gray columns refer to the P1-2020 phase, dark gray columns to P2-2020, and black columns to P3-2020. All the data depicted in this figure are also reported in Table 3, with the *P* values referring to the relative comparisons.

The burden of patients with respiratory failure requiring prompt assistance, hospitalization, or admission to the ICU grew significantly in 2020. Table 3 and Figure 3 report the mean daily values of all the major events considered in this study, grouped by the period of observation. Between P1-2020 and P3-2020, the mean number of patients needing oxygen therapy more than tripled, from 5.3 to 16.7/d; *P* < 0.0001), while that of patients requiring NIV rose from 0.9 to 5.7/d (+575.2%; *P* < 0.0001). Daily admissions from the ED increased by only 23.6% (*P* = 0.0056); however, taking into account the massive decrease in attendance between P1-2020 and P3-2020, the mean percentage of admitted patients (Figure 2; Supplemental Table S3) rose from 13.7% to 46.8% of daily attendance (+240.9%; *P* < 0.0001). Mean daily ICU admissions from the ED rose from 1.0 to 2.5 (+152.2%; *P* = 0.0002), corresponding to an increment from 0.5% to 3.4% of daily attendance (+595.8%; *P* < 0.0001). The overall in-hospital mortality of ED patients in 2020 was 3.4%. However, among COVID-19 ED patients, in-hospital mortality was as high as 17.3% (Table 1), significantly higher than that of 2020 non-COVID patients (2.1%; *P* < 0.0001). The burden of the COVID-19 patients weighed heavily upon 2020 mortality rates, as documented by the remarkable increase in overall in-hospital mortality of ED patients (+133.6% in 2020 compared with 2019, *P* < 0.0001) reported in Table 3.

**TABLE 3:** Mean Daily Values of Major Events (Confirmed COVID-19 Cases, Admissions, Patients Requiring Oxygen Therapy or NIV, in-Hospital Deaths), Grouped and Compared According to the Phase of Occurrence

	2020				2019				**% Change (** ***P*** **Value)**						
	**Overall**	**P1**	**P2**	**P3**	**Overall**	**P1**	**P2**	**P3**	**2020 vs 2019**	**P1-2020 vs P2-2020**	**P2-2020 vs P3-2020**	**P1-2020 vs P3-2020**	**P1-2020 vs P1-2019**	**P2-2020 vs P2-2019**	**P3-2020 vs P3-2019**
Daily covid patients	10.2	0.0	5.5	23.7	NA	NA	NA	NA	NA	NA	+329.3% (**<0.0001**)	NA	NA	NA	NA
Daily hospital admissions from ED	30.7	28.2	28.2	34.8	30.9	31.8	29.8	30.8	-0.6% (**<0.0001**)	-0.1% (0.8033)	+23.4% (**0.0138**)	+23.6% (**0.0056**)	-11.3% (0.0766)	-7.7% (0.4681)	+14.3% (0.0733)
Daily ICU admissions from ED	1.6	1.0	1.1	2.5	1.6	1.7	1.5	1.6	+1.0% (0.9202)	+11.8% (0.8791)	+125.6% (**0.0025**)	+152.2% (**0.0002**)	-41.2% (0.1044)	-24.0% (0.2695)	+56.8% (**0.0315**)
Daily patients on O_2_ therapy	10.1	5.3	7.0	16.7	5.3	6	5.1	4.9	+90.0% (**<0.0001**)	+32.1% (0.0547)	+137.9% (**<0.0001**)	+214.2% (**<0.0001**)	-11.7% (0.4893)	+36.8% (**0.0306**)	+238.9% (**<0.0001**)
Daily patients on NIV	3.0	0.9	1.8	5.7	0.8	0.8	0.8	0.7	+300.0% (**<0.0001**)	+114.5% (**0.0356**)	+214.7% (**<0.0001**)	+575.2% (**<0.0001**)	+13.3% (0.5842)	+121.4% (**0.0293**)	+725.0% (**<0.0001**)
Daily in-hospital deaths	4.3	2.5	4.2	5.9	1.8	2.6	1.5	1.4	+133.6% (**<0.0001**)	+69.4% (**0.0111**)	+38.6% (**0.0255**)	+134.8% (**<0.0001**)	-3.8% (0.8219)	+188.0% (**<0.0001**)	+309.1% (**<0.0001**)
Daily total attendace	130.4	205.2	118.3	74.3	207.0	212.2	203.4	205.3	37.0% **(<0.0001)**	42.3% (**<0.0001**)	37.2% (**<0.0001**)	63.8% (**<0.0001**)	3.3% (0.234)	41.8% (**<0.0001**)	63.8% (**<0.0001**)

***P***-values referred to the comparisons of mean daily values of observed events for each phase were obtained by Mann-Whitney U test.

Abbreviations: NA, not applicable.

Comparing the events observed in P3-2020 with those of P3-2019 (Table 3), we found marked and significant increases in patients needing oxygen (+238.9%; *P* < 0.0001) or NIV (+725.0%; *P* < 0.0001), transfers to ICU (+56.8%; *P* = 0.0315), and in in-hospital mortality (+309.1%; *P* < 0.0001). The mean number of daily admissions increased from 30.8 to 34.8 (+14.3%; *P* = 0.0733), corresponding to a rise from 14.8% to 46.8% of the daily attendance (+215.5%; *P* < 0.0001) (Supplemental Table S3).

In 2020, only 1,377 non-COVID patients were admitted, compared with 1854 in 2019 (−25.7%; *P* < 0.0001). The vast majority of the reduction in admissions (402 of 477; 84%) was concentrated within phases P2 and P3 (Supplemental Table S4).

## DISCUSSION

Our analysis of the events occurring at the OSR ED in February and March 2020 indicated 2 major phenomena. On one hand, a sudden, sharp reduction in ED attendance that began at the onset of P2-2020, immediately after word spread of the first SARS-CoV-2 infection occurring in Italy, and bottomed out once lockdown measures were put into effect. On the other hand, an overwhelming burden of COVID-19 patients requiring urgent treatment and hospitalization that struck the OSR ED during the P3-2020 phase.

Our observations and their relation with local epidemiologic events reflect the dynamics pertaining to both the population’s behavior and the hospital response primed by the SARS-CoV-2 epidemic. Our analysis split the evolution of the COVID-19 emergency in our area into 3 phases: the pre-epidemic (baseline) conditions, the beginning of the outbreak, and, finally, its peak, right after the lockdown. Each of these phases were well-characterized by important changes in the trends of hospital data.

### Phase 1: General Unawareness

Before the onset of the Lombardy outbreak (P1-2020), in light of alarming news regarding the large-scale epidemic spreading in China, Italian health authorities enforced flight restriction policies and (in accordance with the early WHO definition for suspected COVID-19 cases) compulsory quarantine for symptomatic subjects with a history of travel to China, or who had been in contact with a confirmed or probable COVID-19 case, during the 14 d before symptom onset. However, specific strategies for enhancing the provision and supply of medical tools and appliances suitable for the management of contagious diseases and respiratory failure were not publicly mentioned, hospital-based protection measures were not stepped up, and spontaneous actions aimed at self-protection among the local population (such as wearing face masks) were few and far between. At this stage, reflecting the unchanged behavior of the Italian people, patient flow and clinical activity at the OSR ED continued at its usual pace, as borne out by the comparison of data between P1-2020 and P1-2019 (Tables 2 and 3, and Figure 2).

In this first phase, general concern regarding the risk of an outbreak was insufficient to trigger behavioral changes among the population and impact the ordinary attendance and clinical activity in the ED.

### Phase 2: Adaptation

Starting on February 21 (P2-2020), the day after the first autochthonous SARS-CoV-2-positive case was identified in Lombardy, overall ED attendance abruptly decreased by 41.8% when compared with the previous year (Table 2; Figure 2).

This sudden change in the behavior of the local population was a spontaneous response to the concerning news about the first COVID-19 case observed in Lombardy, and occurred before any restrictions to circulation and social activities were put in place by the authorities. Of note, the extent of this spontaneous, early “escape” from the ED accounted for 66% of the total decrease in attendance observed from P1-2020 to P3-2020, with the subsequent decrease after March 8, when lockdown measures were enforced, accounting for the remaining 34% (Figure 2).

The lion’s share of the reduction in ED attendance involved patients presenting for nonspecific complaints, characterized by low rates of complications (skin rashes, ocular symptoms, limb or lumbar pain, etc.), but also involved more potentially ominous presentations, such as chest pain (−36% overall, and -49% among walk-in patients). This suggests that the fear of infection from inside the health-care facilities exceeded the concern over a potentially dangerous symptom, such as chest pain. The 25.7%-drop in the absolute number of non-COVID cases needing hospital admission recorded in 2020 (Supplemental Table S3) is another strong indication that a substantial number of patients with potentially serious conditions other than COVID-19 likely avoided reporting to the ED.

Previous studies regarding the ED patient flow during the 2009 H1N1 pandemic, which was perceived as a low-anxiety event in a large survey from United Kingdom,^6^ documented an important increase in the number of ED visits during the outbreak period, ranging from 18%^7^ to 34%.^8^ On the other hand, a report from Sierra Leone published in 2015 documented a 63% drop in ED visits during the Ebolavirus outbreak.^9^ The reduction in overall attendance (−37%) documented at the OSR ED falls in-between these 2 extremes, and is in line with previous observations made during other coronavirus outbreaks. A report from Hong Kong showed a 24% decrease in all-cause ED visits^10^ during the 2003 SARS epidemic, while another study from Taiwan reported a 33% drop in overall ED attendance.^11^ Another observational study from South Korea estimated a 33% reduction in ED admissions during the 2015 Middle East respiratory syndrome (MERS) outbreak.^12^ Taken together, these data suggest that, in times of epidemic, ED use is strongly influenced by the apparent danger posed by the infection. Notably, while the effects of lockdown on reducing the ED workload took 13 d to kick in, the fear of contagion took hold immediately.

The drop in the OSR ED attendance represented a great advantage in terms of preventing overcrowding, allowing the ED organization and personnel to prepare themselves for the upcoming brunt of infected patients, and to adapt to the specific needs of this new disease gleaned from observation of the first few cases.^13^ During this phase, the OSR ED and wards reorganized their activity and enacted strategies aimed at adapting their response to the SARS-CoV-2 emergency. Ward activities were modified to increase the capacity of care for patients with COVID-19. Elective surgery and out-patient visits were progressively reduced; both medical and surgical wards and a sizable number of staff were shifted to COVID-19 units; new supplementary sub-intensive and ICUs were created for COVID-19 patients.^14^ The ED relocated triage activity outside of the building and created 2 separate pathways for high- and low-risk COVID-19 patients. On February 26, a “Respiratory Medicine” area was set up in the ED, dedicated to suspected and confirmed COVID-19 cases, isolated from the other areas, and equipped for managing the highest possible number of respiratory failure cases. The second phase began as soon as fear of contagion spread among the population, acting as a powerful deterrent in keeping people from reporting to ED.

### Phase 3: Endeavor

In the third phase (P3-2020), the OSR ED, as well as the hospital as a whole, struggled with the massive burden of COVID-19 patients. By this stage, oxygen-equipped ED beds, supplemental oxygen tanks, supplies of ventilators and masks for the delivery of NIV,^15^ available ICU and ward beds dedicated to COVID-19 patients, and even personal protective equipment (PPE) stockpiles were all barely sufficient to satisfy the increasing needs imposed by the surge of COVID-19 patients, despite an extraordinary and relentless hospital provision effort. In the days corresponding to the peak of COVID-19 patient attendance, the fear of reaching a critical depletion of resources was palpable among the ED staff. All our data regarding the P3-2020 phase and its comparison with the P3-2019 phase (Table 3) confirm a massive influx of patients with respiratory failure (+238.9% in the daily number of ED patients requiring oxygen therapy, and +725.0% in the daily number of ED patients requiring NIV), of hospital admission demand (+14.3% in the daily number of admissions from the ED, with +215.5% in admissions among the daily attendance), of intensive treatment need (+56.8% in the daily number of ICU admissions from the ED, or a 332.8% relative increase among the daily attendance).

The unprecedented peak of in-hospital mortality recorded in P3-2020 (+309.1% compared with P3-2019) further attests to the extent of this emergency and its dire consequences.

The third phase is the crux of the epidemic disaster, in which the system preparedness and capacity to surge are tested against the real population’s needs. The events in this phase are heavily influenced by the decisions taken during the preceding 2 phases. In fact, this phase represents the final outcome of complex interactions between the virus’s biological characteristics, the clinical features of the infection, population behavior, and the available health-care resources.

### Direct and Indirect Impacts of COVID-19 on the OSR ED

Major emergencies have both direct and indirect impacts. The direct impact includes morbidity and mortality as consequences of event severity: in these scenarios a portion of deaths and disability is unavoidable, despite optimal treatment.^16^ The direct impact of COVID-19 on our hospital can be clearly appreciated by the dramatic changes in the rates of major clinical events triggered by this epidemic, especially in the P3-2020 phase.

The indirect impact is related to the diversion or depletion of resources from ordinary health-care activities, as well as the tendency of people to avoid reporting to the hospital because of inability to travel and/or fear of contagion inside the health-care facilities.^16^ During the 2014 West Africa Ebolavirus epidemic, lack of routine care for malaria, HIV/AIDS, and tuberculosis led to an estimated fatality rate even greater than that directly attributable to Ebola infection.^17^ During the fall 2009 influenza A (H1N1) pandemic, a higher mortality rate for myocardial infarction in US hospitals was described by Rubinson et al.^8^

During the COVID-19 lockdown, Italian authorities enforced heavy restrictions on people’s movements, banned outdoor activities, and closed parks, restaurants, and any other nonessential social gathering places. These measures may have led to a reduction in the global risk of some acute conditions, such as trauma and myocardial infarction. Nevertheless, lockdown measures per se do not seem to account for the massive drop in ED visits seen in the early phases of the epidemic. Furthermore, we observed an unprecedented reduction in hospital admissions from the ED of non-COVID patients during the COVID-19 epidemic, suggesting that a considerable number non-COVID patients with acute and potentially severe conditions requiring hospital treatment “escaped,” or failed to report to, the ED. All these data strongly suggest that a significant number of missed or delayed diagnoses will have accompanied the COVID-19 wave, worsening these patients’ outcomes. For this reason, the spontaneous collective response of avoiding the ED that we observed should probably be considered a maladaptive one.^18^

The capacity of national health systems to ensure continuity of essential health-care services in times of epidemics is critical to minimizing other potential health emergencies.^19,20^ Nonetheless, our data suggest that its effectiveness could be greatly undermined by a widespread perceived lack of safety in accessing health-care facilities, as an indirect effect of epidemics themselves.

### Lessons Learned

Based on the OSR COVID-19 experience, some specific interventions can be advised for each of the outbreak phases.

During the first phase, flight restriction policies should be coupled with local measures of protection (eg, reasonable use of PPE). In addition, proactive strategies aimed at enhancing local preparedness should be put in place. These include gathering relevant information about the clinical features of the new infection, boosting the provision of materials needed according to the type of infection (eg, in the case of a respiratory coronavirus infection: O2 delivery systems, NIV devices, ventilators), training both the health personnel and the population, devising local protocols and pathways for the isolation and management of infected patients, planning for the possible need of supplementary intensive care resources, and identifying a core group of health-care personnel and resources that could be promptly reassigned to the care of infected patients.

During the second phase, once evidence emerges that the outbreak has overcome the local containment measures based on traditional case identification and contact tracing, all the plans and protocols prepared during the first phase should be activated stepwise, at a pace dictated by the rate of infection spread. Separate pathways for the care of “disaster” patients and “nondisaster” patients should be established. To prevent the ED “escape” phenomenon, clear and reassuring messages must be sent to the population regarding the safety and the low-risk of infection at health-care facilities. The care of ordinary, “nondisaster” patients (in particular for time-dependent conditions like stroke, myocardial infarction, and trauma) must be guaranteed. This can be achieved by sparing adequate resources for “nondisaster” patients in each hospital, or by identifying specific purely “nondisaster hospitals” within the local hospital system.

Duplications and redundancies (eg, multiple triage sites) should be avoided to minimize the dispersion of staff and resources, especially during the peak of the “disaster” patients (third phase). Extra resources of care, such as supplementary ICU beds and staff, should be provided but, ideally, they should be added to the existing structures instead of being built far away as “stand-alone,” new facilities.

## STUDY LIMITATIONS

The retrospective collection of non-COVID patient data could have been biased by possible errors in the recording of data. This is why we chose to limit our analysis to a few clear events, easily retrievable and hardly mistakable. However, this selection prevented us from further in-depth analysis regarding other possible factors (eg, race, comorbidities, social status, etc…) that could have influenced mortality or admissions data. The definition of final diagnoses other than COVID-19 was highly heterogeneous in the electronic records. Hence, we opted not to trace specific final diagnoses and, thus, did not include this aspect in our analysis. Although the definition of the chief complaint at triage was consistently more homogeneous, differences in interpretation of patients’ complaints by triage nurses may have occurred.

## CONCLUSIONS

This is the first report comparing ED use and clinical activity during the SARS-CoV-2 pandemic in Italy with that of the previous year. A 3-phase pattern emerged, describing the dynamics of the interactions among the local population, COVID-19 patients, and ED resources during the exceptional first 2 mo of the 2020 Lombardy outbreak. It seems reasonable to speculate that this small-scale scenario could repeat itself in other, similar epidemiological contexts. Each of these 3 phases presents specific issues that require sensible solutions, and doubtless merit further dedicated analyses.

During major emergencies, avoidable deaths and disabilities depend on the capacity of the health system to maintain the same level of care despite the increased strain, and this capacity to surge is ultimately dependent on preparedness^21,22^ and knowledge. It is our hope that our observations and the lessons drawn from our experience may be instructive in devising new strategies for coping with the direct and indirect impacts of a pandemic emergency.

## Supplementary material

For supplementary material accompanying this paper visit https://doi.org/10.1017/dmp.2020.265. click here to view supplementary material
