# Supplementary material for: Stranger Months: How SARS-CoV-2, Fear of Contagion, and Lockdown Measures Impacted Attendance and Clinical Activity During February and March 2020 at an Urban Emergency Department in Milan
Source: Disaster Med Public Health Prep. 2020 Jul 27:1–10. doi: 10.1017/dmp.2020.265 (PMC7588723; doi:10.1017/dmp.2020.265)
Supplement: Supplementary file 1 [file S1935789320002657sup001.docx]

**SUPPLEMENTARY MATERIAL**

**Table S1. Charts of the 20 most frequent chief complaints reported by patients at triage, in the 2020 and 2019 60-day periods, stratified by modality of presentation.**

| **2020** | | | | | | | **2019** | | | | | | |
| --- | --- | --- | --- | --- | --- | --- | --- | --- | --- | --- | --- | --- | --- |
| **MAIN COMPLAINT** | **SP** | **TBA** | **TOT** | **SP** | **TBA** |  | **MAIN COMPLAINT** | **SP** | **TBA** | **TOT** | **SP** | **TBA** | **TOT** |
| 1. TRAUMA | 699 | 250 | 949 | 15.31% | 12.61% | 14.50% | 1. TRAUMA | 1633 | 448 | 2081 | 19.24% | 23.97% | 20.09% |
| 2. ABDOMINAL PAIN | 566 | 120 | 686 | 12.40% | 6.05% | 10.48% | 2. ABDOMINAL PAIN | 1091 | 129 | 1220 | 12.85% | 6.90% | 11.78% |
| 3. FEVER | 491 | 339 | 830 | 10.76% | 17.10% | 12.68% | 3. CHEST PAIN | 411 | 127 | 538 | 4.84% | 6.80% | 5.20% |
| 4. DYSPNEA | 304 | 428 | 732 | 6.66% | 21.59% | 11.18% | 4. OCULAR SYMPTOMS | 490 | 4 | 494 | 5.77% | 0.21% | 4.77% |
| 5. CHEST PAIN | 209 | 134 | 343 | 4.58% | 6.76% | 5.24% | 5. FEVER | 463 | 85 | 548 | 5.46% | 4.55% | 5.29% |
| 6. NEUROLOGIC SYMPTOMS | 98 | 121 | 219 | 2.15% | 6.10% | 3.35% | 6. DYSPNEA | 271 | 162 | 433 | 3.19% | 8.67% | 4.18% |
| 7. CHOUGH | 145 | 61 | 206 | 3.18% | 3.08% | 3.15% | 7. NEUROLOGIC SYMPTOMS | 189 | 172 | 361 | 2.23% | 9.20% | 3.49% |
| 8. OCULAR SYMPTOMS | 202 | 4 | 206 | 4.42% | 0.20% | 3.15% | 8. HEADACHE | 223 | 49 | 272 | 2.63% | 2.62% | 2.63% |
| 9. INFERIOR LIMB PAIN | 118 | 16 | 134 | 2.58% | 0.81% | 2.05% | 9. INFERIOR LIMB PAIN | 248 | 25 | 273 | 2.92% | 1.34% | 2.64% |
| 10. HEADACHE | 107 | 24 | 131 | 2.34% | 1.21% | 2.00% | 10. SKIN RASH/SKIN ALLERGIC REACTION | 223 | 12 | 235 | 2.63% | 0.64% | 2.27% |
| 11. PALPITATIONS/ARRHYTHMIAS | 84 | 36 | 120 | 1.84% | 1.82% | 1.83% | 11. SYNCOPE/PRESYNCOPE | 78 | 123 | 201 | 0.92% | 6.58% | 1.94% |
| 12. SKIN RASH/SKIN ALLERGIC REACTION | 88 | 7 | 95 | 1.93% | 0.35% | 1.45% | 12. LUMBAR PAIN | 155 | 24 | 179 | 1.83% | 1.28% | 1.73% |
| 13. EAR PAIN | 89 | 0 | 89 | 1.95% | 0.00% | 1.36% | 13. PALPITAZIONI/ARITMIA | 155 | 27 | 182 | 1.83% | 1.44% | 1.76% |
| 14. LUMBAR PAIN | 72 | 11 | 83 | 1.58% | 0.55% | 1.27% | 14. CHOUGH | 153 | 13 | 166 | 1.80% | 0.70% | 1.60% |
| 15. VERTIGO | 60 | 27 | 87 | 1.31% | 1.36% | 1.33% | 15. EAR PAIN | 160 | 2 | 162 | 1.89% | 0.11% | 1.56% |
| 16. WOUND/INFECTED WOUND | 73 | 8 | 81 | 1.60% | 0.40% | 1.24% | 16. WOUND/INFECTED WOUND | 130 | 12 | 142 | 1.53% | 0.64% | 1.37% |
| 17. HEMATURIA | 57 | 7 | 64 | 1.25% | 0.35% | 0.98% | 17. VERTIGO | 98 | 43 | 141 | 1.15% | 2.30% | 1.36% |
| 18. SUPERIOR LIMB PAIN | 56 | 2 | 58 | 1.23% | 0.10% | 0.89% | 18. SUPERIOR LIMB PAIN | 130 | 7 | 137 | 1.53% | 0.37% | 1.32% |
| 19. ABSCESS | 51 |  | 51 | 1.12% | 0.00% | 0.78% | 19. ABSCESS | 97 | 3 | 100 | 1.14% | 0.16% | 0.97% |
| 20. SYNCOPE/PRESYNCOPE | 43 | 118 | 161 | 0.94% | 5.95% | 2.46% | 20. DIARRHEA | 77 | 21 | 98 | 0.91% | 1.12% | 0.95% |
| **TOTAL ATTENDANCES** | **4565** | **1982** | **6547** | **100%** | **100%** | **100%** | **TOTAL ATTENDANCES** | **8487** | **1869** | **10356** | **100%** | **100%** | **100%** |

Data from the pediatric and obstetric/gynecologic area were not included due to a different registration procedure. SP= self presented. TBA= transported by ambulance.

**Table S2. Absolute and percentage change of the most frequent complaints reported by patients at triage in the 2020 as compared to 2019, stratified by modality of presentation.**

| **MAIN COMPLAINT** | **2020** | | | **2019** | | | **ABSOLUTE CHANGE** | | | **% CHANGE** | | |
| --- | --- | --- | --- | --- | --- | --- | --- | --- | --- | --- | --- | --- |
|  | **SP** | **TBA** | **ALL** | **SP** | **TBA** | **ALL** | **SP** | **TBA** | **ALL** | **SP** | **TBA** | **ALL** |
| **TRAUMA** | 699 | 250 | 949 | 1633 | 448 | 2081 | -934 | -198 | -1132 | -57.2% | -44.2% | -54.4% |
| **ABDOMINAL PAIN** | 566 | 120 | 686 | 1091 | 129 | 1220 | -525 | -9 | -534 | -48.1% | -7.0% | -43.8% |
| **FEVER** | 491 | 339 | 830 | 463 | 85 | 548 | 28 | 254 | 282 | 6.0% | 298.8% | 51.5% |
| **DYSPNEA** | 304 | 428 | 732 | 271 | 162 | 433 | 33 | 266 | 299 | 12.2% | 164.2% | 69.1% |
| **CHEST PAIN** | 209 | 134 | 343 | 411 | 127 | 538 | -202 | 7 | -195 | -49.1% | 5.5% | -36.2% |
| **NEUROLOGIC SYMPTOMS** | 98 | 121 | 219 | 189 | 172 | 361 | -91 | -51 | -142 | -48.1% | -29.7% | -39.3% |
| **CHOUGH** | 145 | 61 | 206 | 153 | 13 | 166 | -8 | 48 | 40 | -5.2% | 369.2% | 24.1% |
| **OCULAR SYMPTOMS** | 202 | 4 | 206 | 490 | 4 | 494 | -288 | 0 | -288 | -58.8% | 0.0% | -58.3% |
| **INFERIOR LIMB PAIN** | 118 | 16 | 134 | 248 | 25 | 273 | -130 | -9 | -139 | -52.4% | -36.0% | -50.9% |
| **HEADACHE** | 107 | 24 | 131 | 223 | 49 | 272 | -116 | -25 | -141 | -52.0% | -51.0% | -51.8% |
| **PALPITATIONS/ARRHYTHMIAS** | 84 | 36 | 120 | 155 | 27 | 182 | -71 | 9 | -62 | -45.8% | 33.3% | -34.1% |
| **SKIN RASH/SKIN ALLERGIC REACTION** | 88 | 7 | 95 | 223 | 12 | 235 | -135 | -5 | -140 | -60.5% | -41.7% | -59.6% |
| **EAR PAIN** | 89 | 0 | 89 | 160 | 2 | 162 | -71 | -2 | -73 | -44.4% | -100.0% | -45.1% |
| **LUMBAR PAIN** | 72 | 11 | 83 | 155 | 24 | 179 | -83 | -13 | -96 | -53.5% | -54.2% | -53.6% |
| **VERTIGO** | 60 | 27 | 87 | 98 | 43 | 141 | -38 | -16 | -54 | -38.8% | -37.2% | -38.3% |
| **WOUND/INFECTED WOUND** | 73 | 8 | 81 | 130 | 12 | 142 | -57 | -4 | -61 | -43.8% | -33.3% | -43.0% |
| **HEMATURIA** | 57 | 7 | 64 | 79 | 9 | 88 | -22 | -2 | -24 | -27.8% | -22.2% | -27.3% |
| **SUPERIOR LIMB PAIN** | 56 | 2 | 58 | 130 | 7 | 137 | -74 | -5 | -79 | -56.9% | -71.4% | -57.7% |
| **ABSCESS** | 51 | 0 | 51 | 97 | 3 | 100 | -46 | -3 | -49 | -47.4% | -100.0% | -49.0% |
| **SYNCOPE/PRESYNCOPE** | 43 | 118 | 161 | 78 | 123 | 201 | -35 | -5 | -40 | -44.9% | -4.1% | -19.9% |
| **DIARRHEA** | 30 | 15 | 45 | 77 | 21 | 98 | -47 | -6 | -53 | -61.0% | -28.6% | -54.1% |
| **TOTAL ATTENDANCES** | **4565** | **1982** | **6547** | **8487** | **1869** | **10356** | **-3922** | **113** | **-3809** | **-46.2%** | **6.0%** | **-36.8%** |

Data from the pediatric and obstetric/gynecologic area were not included due to a different registration procedure. SP= self presented. TBA= transported by ambulance.

**Table S3. Mean proportion (percentage) of observed major events (confirmed COVID-19 cases. Admissions, patients requiring oxygen therapy or NIV, in-hospital deaths) in relation to the relative mean total daily attendance, stratified and compared according to the phase of occurrence.**

|  | **2020** | | | | **2019** | | | | **% CHANGE (p value)** | | | | | | |
| --- | --- | --- | --- | --- | --- | --- | --- | --- | --- | --- | --- | --- | --- | --- | --- |
|  | **OVERALL** | **P1** | **P2** | **P3** | **OVERALL** | **P1** | **P2** | **P3** | **2020 vs 2019** | **P1-2020 vs P2-2020** | **P2-2020 vs P3-2020** | **P1-2020 vs P3-2020** | **P1-2020 vs P1-2019** | **P2-2020 vs P2-2019** | **P3-2020 vs P3-2019** |
| **DAILY COVID PATIENTS** | 7.8% | 0.0% | 4.7% | 31.9% | NA | NA | NA | NA | NA | NA | +489.2% (**<0.0001**) | NA | NA | NA | NA |
| **DAILY HOSPITAL ADMISSIONS FROM ED** | 23.5% | 13.7% | 23.8% | 46.8% | 14.9% | 15.0% | 15.0% | 14.8% | +57.7% (**<0.0001**) | +73.6% (**<0.0001**) | +96.4% (**<0.0001**) | +240.9% (**<0.0001**) | -8.3% (0.6298) | +58.7% (**<0.0001**) | +215.5% (**<0.0001**) |
| **DAILY ICU ADMISSIONS FROM ED** | 1.2% | 0.5% | 0.9% | 3.4% | 0.8% | 0.8% | 0.7% | 0.8% | +50.0% (**<0.0001**) | +93.8% (**0.0485**) | +295.0% (**<0.0001**) | +595.8% (**<0.0001**) | -39.2% (0.10223) | +30.7% (0.3759) | +332.8% (**<0.0001**) |
| **DAILY PATIENTS ON O_2_ THERAPY** | 7.8% | 2.6% | 5.9% | 22.4% | 2.6% | 2.8% | 2.5% | 2.4% | +200.0% (**<0.0001**) | +129.1% (**<0.0001**) | +278.5% (**<0.0001**) | +767.0% (**<0.0001**) | -8.7% (0.4911) | +135.2% (**<0.0001**) | +835.7% (**<0.0001**) |
| **DAILY PATIENTS ON NIV** | 2.3% | 0.4% | 1.5% | 7.7% | 0.4% | 0.4% | 0.4% | 0.3% | +475.0% (**<0.0001**) | +272.1% (**<0.0001**) | +400.8% (**<0.0001**) | +1763.1% (**<0.0001**) | +17.2% (0.6532) | +280.8% (**<0.0001**) | +2177.7% (**<0.0001**) |
| **DAILY IN-HOSPITAL DEATHS** | 3.4% | 1.2% | 3.6% | 7.9% | 0.9% | 1.2% | 0.7% | 0.7% | +270.9% (**<0.0001**) | +193.8% (**<0.0001**) | +120.5% (**<0.0001**) | +547.8% (**<0.0001**) | -0.6% (0.9770) | +395.2% (**<0.0001**) | +1029.4% (**<0.0001**) |

P-values were obtained by the chi square test. NA = not applicable.

**Table S4. Absolute and relative differences in admissions regarding non-COVID patients in the whole 2020 and 2019 time-windows. and in the P2+P3 phases. P values were obtained by Mann-Whitney U test comparing the distributions of daily admissions.**

| **non-COVID ADMISSIONS** | **ABSOLUTE NUMBER** | **DIFFERENCE** | **% CHANGE** | **p value** |
| --- | --- | --- | --- | --- |
| **2020** | 1377 | -477 | -25.7 | <0.0001 |
| **2019** | 1854 |  |  |  |
| **P2+P3 2020** | 817 | -402 | -33.0 | <0.0001 |
| **P2+P3 2019** | 1219 |  |  |  |
